# Prion domains as a driving force for the assembly of functional nanomaterials

**DOI:** 10.1080/19336896.2020.1785659

**Published:** 2020-06-28

**Authors:** Weiqiang Wang, Salvador Ventura

**Affiliations:** Institut de Biotecnologia i de Biomedicina and Departament de Bioquímica i Biologia Molecular, Universitat Autònoma de Barcelona, Bellaterra (Barcelona), Spain

**Keywords:** Prions, amyloids, self-assembly, prion-like domains, nanomaterials, yeast

## Abstract

Amyloids display a highly ordered fibrillar structure. Many of these assemblies appear associated with human disease. However, the controllable, stable, tunable, and robust nature of amyloid fibrils can be exploited to build up remarkable nanomaterials with a wide range of applications in biomedicine and biotechnology. Functional prions constitute a particular class of amyloids. These transmissible proteins exhibit a modular architecture, with a disordered prion domain responsible for the assembly and one or more globular domains that account for the activity. Importantly, the original globular protein can be replaced with any protein of interest, without compromising the fibrillation potential. These genetic fusions form fibrils in which the globular domain remains folded, rendering functional nanostructures. However, in some cases, steric hindrance restricts the activity of these fibrils. This limitation can be solved by dissecting prion domains into shorter sequences that keep their self-assembling properties while allowing better access to the active protein in the fibrillar state. In this review, we will discuss the properties of prion-like functional nanomaterials and the amazing applications of these biocompatible fibrillar arrangements.

## Introduction

Amyloid proteins can self-assemble into ordered fibrillar structures. These amyloid fibrils, constitute a hallmark of human amyloidosis as well as of neurodegenerative diseases, including Alzheimer’s, Parkinson’s, Type Ⅱ diabetes, and others [1–3]. The discovery of functional amyloids in different organisms, from bacteria to humans, assisting different physiological functions in living cells [4], has changed the amyloid/pathogenesis paradigm. For instance, functional amyloids are involved in curli-mediated biofilm formation in *E. coli* [5], memory persistence in *Drosophila* [6], hypersensitive response activation in plants [7], melanin polymerization in mammalian cells [8], and hormone storage in humans [9]. Amyloid fibrils display a remarkably similar quaternary structure, formed by the assembly of monomers into a characteristic ‘cross-β’ structure [10], resulting from the stacking of intermolecular β-sheets arrayed perpendicular to fibrils axis, which are stabilized by numerous hydrogen bonds between neighbouring strands [11], together with π-π and hydrophobic interactions [12]. Such a unique spatial structure endows amyloid fibrils with high stability and high resistance to extreme physicochemical conditions, such as treatment with proteinases, chaotropic agents, and high temperatures[13]. These properties make amyloid fibrils ideal building blocks for designing novel protein/peptide self-assembled nanostructures. Indeed, amyloid fibrils have been incorporated as structural components in functional materials with different biomedical and biotechnological applications, including nanodevices [14], biomembranes [15], hydrogels [16], biosensors [17], and energy conversion materials [18]. However, the inherent conformational transition towards a β-sheet rich structure during fibrillation necessarily implies the loss of the globular native fold and the subsequent inactivation of the protein. Therefore, designing fibrillar structures containing correctly folded and active proteins is still a challenging task. A way to bypass this limitation is by designing a hybrid structure in which the globular proteins are chemically attached to preformed amyloid fibrils [19]. However, this approach is restrained by a reduced protein conjugating chemistry, undesired cross-reactivity between the polypeptides, and the unavoidable inactivation of a fraction of the protein during conjugation.

Prions are proteins able to switch between two or more conformations, of which at least one corresponds to an amyloid fold [20–22]. So far, only a prion protein (PrP) has identified in mammals; PrP is well conserved across species and linked to different neurological pathologies [23]. Roughly ten prion proteins have been characterized in the yeast *Saccharomyces cerevisiae*. They are usually referred to as functional prions since, in contrast to ‘toxic’ mammalian prions, they do not seem to have a significant impact on viability, but instead, they play physiological roles, especially in the adaptation to changing extracellular environments [24,25]. They usually consist of an intrinsically unstructured region of low complexity enriched in asparagine (N) and glutamine (Q) residues, known as the prion domain (PrD), accompanied by one or more globular domains [26,27]. The PrD is both necessary and sufficient for self-assembly, whereas the globular domain accounts for the protein functionality [28]. Yeast prions are thus modular and, in principle, the original globular domain can be replaced with any desired functional protein without impacting the fibrillation propensity significantly. Domains with properties resembling those of PrDs have been identified in the proteins of different organisms, including humans, and are named prion-like domains (PrLDs).

In contrast to the fast and challenging to control aggregation reactions of pathogenic amyloids, prion and prion-like proteins generally display slow and tunable aggregation kinetics, which are considered optimal for the design of self-assembling materials [29]. Actually, different enzymes such as alkaline phosphatase (AP) [30], carbonic anhydrase (CA) [31], methyl-parathion hydrolase (MPH) [32] or other globular proteins like protein G and the Z domain [33] have been genetically fused to the PrDs of yeast prion Sup35 or Ure2p to generate functionalized nanomaterials in which the PrDs constitute the spine of the fibrils, whereas the globular domains hang from them in a folded and functional conformation. These materials are biocompatible, degradable, and environmentally friendly and can be employed in applications encompassing from catalytic nanowires [30] to nanosensors [32]. Here, we summarize the features of functional prions in yeast and review the use of PrDs and PrLDs to generate genetically encoded functionalized nanomaterials and discuss their potential applications.

## Functional prions in yeast

The concept of infectious proteins was initially proposed to lie behind different neurological diseases, including Creutzfeldt-Jacob disease in humans, sheep scrapie and bovine spongiform encephalopathy, all caused by the amyloid state of the prion protein (PrP) [34–36]. Thus, prions were assumed to be pathogenic agents without any beneficial functional implication.

Initially, the phenotypes [PSI+] and [URE3] of yeast were identified as non-chromosomal genetic elements. Later on, Aigle and Lacroute reported that *ure2* nuclear gene mutant strains were unable to propagate the [URE3] element but manifested the same phenotype as [URE3] cells, namely de-repression of nitrogen catabolism genes[37]; however, they did not realize that [URE3] could be a prion of Ure2p. It was Reed B. Wickner, who, based on these pieces of evidence and his own experiments, recognized that the relationship between *ure2* and [URE3] was not that expected for a chromosomal gene necessary for the propagation of a nucleic acid replicon, but instead that [URE3] was an inactive form of Ure2p able to inactivate the normal protein form, acting thus as a prion [38,39]. Then his group inferred that this could also be true for the [PSI+] phenotype [38], and concluded that previous studies [40–42] supported [PSI+] being a prion of Sup35p. Moreover, they established a series of genetic criteria that identify yeast prions [43]: i) a reversible curability, ii) the phenotype appearance is induced by overexpression of the prion protein, and iii) the phenotype resembles that of a prion protein gene recessive mutation.

These genetic criteria were useful for the discovery of a range of new prions including [PIN+] [44], [SWI+] [45], [MOT+] [46] and [MOD+] [24]. The formation of amyloid *in vitro* by Sup35p [47], and Ure2p [48], the protease resistance of Ure2p in [URE3] strains [49], together with the seeding properties of cellular extracts in [PSI+] [50], all converged to indicate that the prion form of these proteins was an amyloid. This assumption was further confirmed after observing that *in vitro* formed Sup35p amyloids transmit the [PSI+] trait [51].

**Figure 1.**
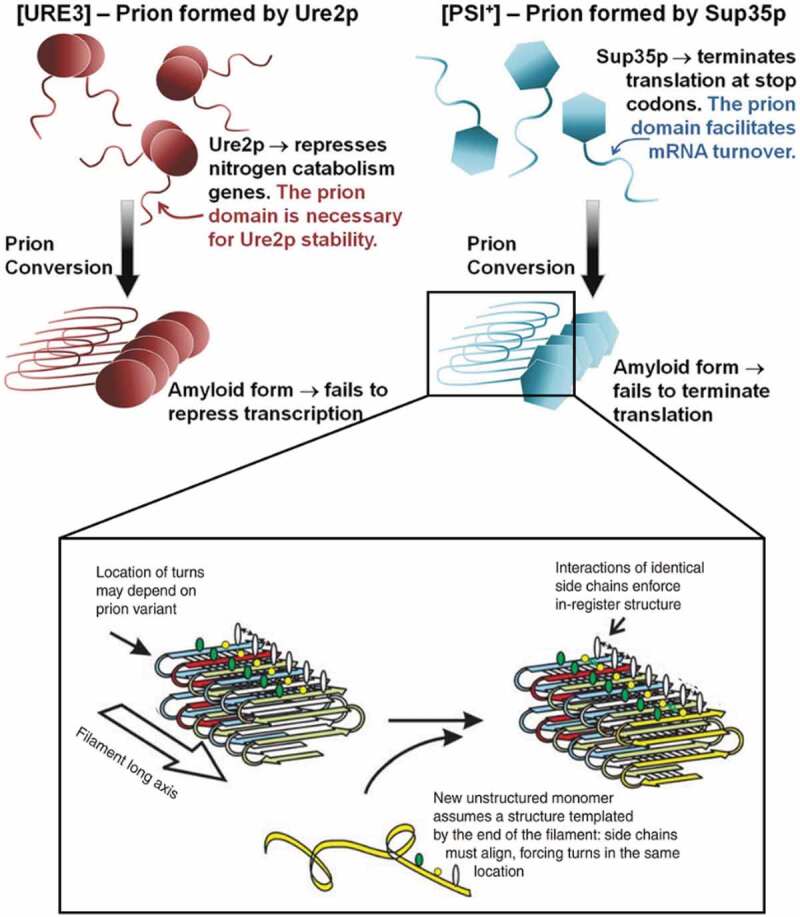
Typical prions [URE3] and [PSI+] of S. cerevisiae. These prions rely on self-propagating amyloids of Ure2p and Sup35p, respectively. The insert is the proposed mechanism of conformational templating by the prion domain. Energetically favourable interactions between identical side chains enforce the in-register architecture of these amyloids. A new monomer being added to the end of the filament must assume the same conformation as that of molecules previously incorporated into the filament. (adapted with permission).[43]

The inheritance of phenotypic traits due to prion formation involves a structural conversion, which in a majority of cases is driven by a Q/N rich PrD [52]. PrDs proved to be sufficient to induce and propagate the prion state. They become integrated into the core of the amyloid structure, which is thought to be arranged as an in-register parallel β-sheet architecture [10], according to nuclear magnetic resonance (NMR) and electron microscopy studies of the amyloids of Sup35p and Ure2p PrDs (Figure 1) [53,54]. The globular domains appended to the PrDs might retain their native structure in the amyloid filaments (Figure 1), although the activity of these domains was found to be somehow reduced, likely because of steric impediments [31]. Such folded, in-register parallel β-sheet architecture perfectly explains the mechanism of protein templating in prion conformational conversion (Figure 1) [55]. In particular, the favourable interactions between hydrophobic or hydrophilic side chains along the long axis of the filament maintains the in-register β-sheet structure. The same interactions force the new monomer to join the end of the filament to adopt the same conformation as the molecules already in the fibril, just as nucleic acids template their sequences [39]. An exception to this rule is the HET-s prion, from the filamentous fungi *Podospora anserine*, involved in heterokaryon incompatibility, protecting cells from fungal viruses [56]. The amyloid formed by the PrD of HET-s displays a β-helix structure, in which each monomer contributes two turns to the β-helix [57]. Recently, cryo-EM and computational studies suggest that PrP^Sc^ would adopt a similar β-solenoid structure [58,59]. In fungal prions, the self-propagation of the prion phenotype during cell division is generally accomplished by the action of the chaperone system, which fragments existing fibrils, generating seeds that initiate the polymerization reaction in daughter cells [60,61].

The first and most studied of yeast prion proteins are Sup35p and Ure2p (Figure 1), involved in translation termination and nitrogen catabolism regulation, respectively [38]. Sup35p, a subunit of the translation termination factor, has a clear three-domain structure. The N-terminal residues 1–123 (Sup35 N) are sufficient to induce and propagate the [PSI+] prion phenotype and thus constitute the PrD. The C-terminal residues 254–685 (Sup35 C), fold into a globular domain, responsible for the translation termination function [62,63]. The charged middle domain (Sup35 M), corresponding to residues 124 to 253, is important but not required for [PSI+] appearance and maintenance [64,65]. Sup35 N drives self-assembly of Sup35p into an amyloid, which results in a substantial reduction of its translation termination function and leads to an increase of nonsense codon readthrough. The PrD of Sup35p has itself a non-prion function, facilitating mRNA turnover. Ure2p has an architecture similar to that of Sup35p, including an N-terminal PrD (residues 1–65) responsible for [URE3] formation [66] and a C-terminal domain (Ure2 C) structurally similar to glutathione transferase, but involved in repression of nitrogen catabolism genes by binding to the transcription factor Gln3p, an interaction that is significantly impeded in the amyloid state [67].

The modular structure of yeast prions allows to dissect them in order to assess the prionogenic potential of other proteins and, in this way, identify new prions. This is performed by replacing the PrD or globular domain, via genetic engineering, with any desired polypeptide or reporter protein, such as the green fluorescent protein (GFP), to study the ability of the fusion protein to induce [PSI+]-like appearance or to visualize protein inclusions formation in cells [68]. These studies demonstrated that yeast prions could be easily engineered, immediately suggesting that they can be exploited in the design of self-assembling functionalized materials.

## Prion-like nanomaterials obtained via genetic protein fusion

**Figure 2.**
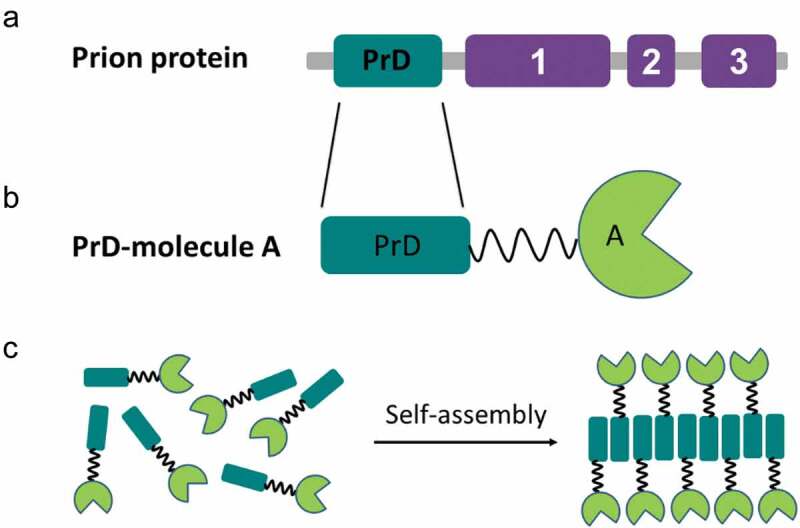
The general genetic fusion approach for functionalized nanomaterials. (a) Domain organization of the prion protein: soft amyloid core (SAC) (red) within the prion domain (PrD) (green) and the respective functional domains (purple) in a prion protein are shown. (b) Cartoon of the prion domain fused to molecule A, which represents a globular functional protein. (c) Soluble fusion protein self-assembly into nanofibrils, which preserve the function of molecule A.

It has been reported that the yeast prion Ure2p exhibits glutathione peroxidase activity in both the native and fibrillar forms [69]. Besides, exogenous globular domains fused to the Ure2p PrD retain a native-like fold and show catalytic activity within *in vitro* formed amyloid filaments [31]. This ability to combine amyloid and globular folds in the aggregated state seems to be generic of yeast prions since the analysis of Sup35p amyloid filaments by cryo-EM, STEM [70], and solid state NMR [71], revealed that filaments of full length Sup35p indicated a thin fibril backbone surrounded by globular C-domains. The formation of a spatially defined fibrillar backbone and the segregation of folded globular domains are central for the design of prion-like self-assembling functionalized nanomaterials, with functions that include enzymatic catalysis [30], biosensing [32], and protein binding [33]. The general strategy to obtain the building blocks for such materials consists of the genetic fusion of a yeast prion PrD to a protein of interest and its recombinant production in microbial cell factories (Figure 2). The purified soluble fusion protein can self-assemble spontaneously into nanofibrils, which exhibit the function of the appended globular domain, under appropriate conditions of temperature, pH, and agitation, in some instances, requiring the presence of detergents.

### Biosensors

The first prion-like nanomaterial obtained via genetic protein fusion was intended to work as a biosensor. Dong Men et al. [32] generated bifunctional protein nanowires (bFPNw) that displayed two different globular proteins: protein G and methyl-parathion hydrolase (MPH), which provide antibody binding and catalytic activities, respectively. They aimed to obtain fibrils with a high enzyme to protein G ratio. Towards this objective, both protein G and MPH were fused individually to the N-terminus of the Sup35p PrD (residues 1–61) and expressed as soluble monomers in *E.coli*. The fibrils formed by the self-assembly of the Sup35^1−61^-protein G fusion were broken into small fragments to be used as seeds. The bFPNw exhibiting two different functions with different ratios were obtained by mixing these seeds and soluble Sup35^1−61^-MPH in different proportions; this also allowed to tune the length of the fibrils. The biological activity of these bFPNw was tested by a typical ELISA assay, implementing them instead of the classical enzyme-conjugated secondary antibody. As a general trend, the longer the nanowires were, the higher the signal amplification they provided. In that work, optimized nanowires of 500 nm in length (ratio of protein G to MPH was 1:8) showed a sensitivity for the detection of the F1 protein from *Yersinia pestis* 1000-fold higher than that of a protein G-MPH fusion and 100-fold higher than that of the traditional HRP-conjugated antibody-based ELISA.

In subsequent work, the same group designed auto-biotinylated bFPNw by introducing a biotin avidin binding system, which can efficiently attach any avidin-labelled commercial enzyme, such as HRP or AP, to the nanowires backbone. This skips the need to produce and purify a different fusion for each intended application[72]. In particular, the biotin acceptor peptide tag (BAP) and the IgG-binding domain, C1, of protein G from *Streptococcus* (SPG) were individually fused to the Sup35p PrD segment described above. The construct Sup35^1−61^-BAP was co-expressed in *E.coli* with another construct encoding for the biotin holoenzyme synthetase (BirA). In this way, the BAP tag was biotinylated by BirA during the expression of the Sup35^1−61^-BAP fusion. Instead of inducing the formation of bifunctional nanowires by seeding, which they found reduced the IgG binding activity of protein G because of the sonication processes, on this occasion, they just mixed the pre-formed biotinylated Sup35^1−61^-BAP fibrils with the Sup35^1−61^-SPG monomer, which was templated and incorporated at the end of the fibrils. The function of the bFPNw was evaluated by performing a typical ELISA in the presence of Streptavidin-HRP instead of the typical secondary IgG-HRP conjugate. For the detection of *Y. pestis* F1 antigen, the auto-biotinylated bFPNw showed higher signal intensity, lower background noise, and better signal stability than that of previous seeding promoted bFPNw. More importantly, they showed 2000- to 4000-fold higher sensitivity than that of a conventional ELISA.

The lessons learned in these two previous works were exploited to construct a highly sensitive fluorescent molecular biosensor. First, an F64 L/S65 T/T203Y/L231 H green fluorescent protein mutant (E^2^GFP) was fused to MPH [73], E^2^GFP shows distinct excitation and emission spectra depending on the pH, which makes it an effective ratiometric pH indicator. The chimeric E^2^ GFP-MPH protein allowed the detection of the pesticide methyl parathion (MP) by sensing the H^+^ ions released during enzymatic reaction. Interestingly enough, when this chimeric protein was further fused to the Sup35p PrD and incorporated into fibrils through its self-assembly, the obtained nanowires showed dramatic enhancement of sensitivity and allowed detection of MP at a concentration as low as 1pmol mL^−1^, which was about 10,000-fold that of the soluble fusion protein biosensor E^2^GFP-MPH and 10^7^ -fold higher than the one of an equimolar mixture of free E^2^GFP and MPH moieties. These nanostructures might become tools for the detection of pesticides in the food industry.

### Enzyme immobilization

Immobilization can enhance the value and usage of commercial enzymes, providing advantages such as an easy separation of the product and the reuse of the enzyme. The lab of Sarah Perrett has recently described PrD-based enzyme immobilization systems [30]. On this occasion, it was the PrD of Ure2p that was exploited to drive the self-assembly, and two different enzymes, AP and HRP, were individually fused to it. Both fusion proteins formed active nanofibrils, where the enzymes become immobilized. Although the fibrils exhibited slightly lower activity than that of the soluble wild type proteins, they could be recycled both in a conventional enzymatic setup and in the context of a continuous microreactor. In a follow up study, the authors encapsulated the soluble PrD-AP protein fusion into a uniform droplet using microfluidic techniques [74]. The encapsulated soluble protein spontaneously self-assembled into a three-dimensional fibrillar network within the droplet, immobilizing AP molecules in their active form. As a result, a catalytically active microgel with a porous architecture was obtained. These hydrated functional nanoparticles, when combined with microfluidics, are ideal entities to implement microscale analytical assays.

### Bioelectrodes

Vincent Forge and colleagues designed a protein-only redox biofilm inspired by the architecture of bacterial electroactive biofilms [75]. Briefly, the formation of the nanowires was induced by the self-assembly of the PrD of HET-s *P. anserine* prion, which was genetically fused to rubredoxin (Rd). This redox protein acts as an electron carrier. The formation of the fibrillar structures allowed the alignment of Rd along the fibril axis in an array, which facilitates the transportation of electrons through the network resulting in an exceptional conductivity. As a proof of concept, such redox active fibrils were successfully applied to mediate the electron transfer towards the multi-cooper enzyme laccase for catalytic oxygen reduction. Therefore, these protein fibrils are expected to find applications as nanometric bioelectrodes.

### Antibody purification

**Figure 3.**
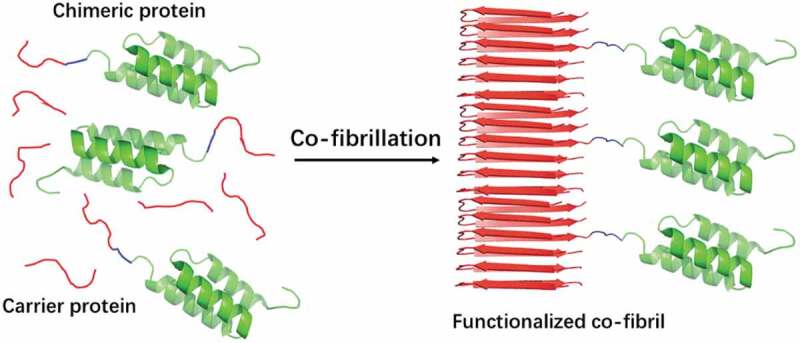
Schematic illustration of co-fibrillation to immobilize globular domains into amyloid fibrils with defined doping frequencies.

The increasing demand of antibodies for diagnostic and therapeutic purposes requests cost efficient means for their purification. In recent work, Torleif Härd and co-workers exploited the small size and high affinity to IgG of the Z-domain from staphylococcal protein A to build up antibody capturing fibrils. A Z-domain dimer (ZZ) was fused to the PrD of either Sup35p (Sup35-ZZ) or Ure2p (ZZ-Ure2). Both of them promoted the fibrillation of the chimeric protein [33]. These fibrils bind to IgG, but their loading capacity was limited by steric hindrance. The PrDs of Sup35p and Ure2p were then co-fibrillated with chimeric Sup35-ZZ and ZZ-Ure2 proteins, respectively, to maximize the accessibility of antibodies to ZZ. This fibril doping strategy resulted in co-fibrils in which ZZ globular domains are more spaced in the fibrillar structure, thus maximizing the fibrils binding capacity (Figure 3). Different doping frequencies were assayed, and in the optimal PrD:protein fusion ratio, the resulting fibrils yielded a capture capacity of 1.8 mg of antibody per mg fibrils, which is 20-fold higher than the one of commercial protein A agarose.

Despite the exceptional binding capacity of ZZ functionalized fibrils, this material should be produced and engineered at large scale before it can commercially compete with the present well positioned methods for purification of antibodies. Towards this objective, the Härd lab integrated both the Sup35-PrD and the chimeric Sup35-ZZ genes in the genome of *Komagataella pastoris* [76]. This yeast continuously expressed and secreted both proteins into the extracellular medium, where they co-fibrillated into ready-to-use functionalized nanofibrils, with an impressive production yield of 35 mg/L culture. Importantly, the separation of the fibrillar material from the cell culture required only centrifugation and their resuspension in a saline buffer. The high yield, high homogeneity, and high stability of the purified material, together with minimal equipment requirement and the low hands on time of this strategy, allows foreseeing, for the first time, the possibility of producing functional prion-like fibrils at an industrial scale.

The chimeric protein consists of a prion-like domain (red) and a Z domain (green, antibody binding domain from Protein A, PDB 1Q2 N), linked via a flexible linker (blue). The chimeric protein is fibrillated with the carrier protein (the prion-like domain alone), in order to allow enough space between globular domains in the fibrils. This reduces steric constraints and maximizes the functionality.

### Biocatalytic cascades

As discussed above, the chemistry-free covalent enzyme immobilization and the high surface to volume ratio of prion-based fibrils make of these nanostructures optimal recyclable catalysers. However, many industrially relevant processes require two or more coupled reactions, and, in most cases, only single-enzyme functionalized fibrils have been characterized. Mats Sandgren and co-workers addressed this issue by building up a catalytic cascade by genetically fusing xylanase A, β-xylosidase, and aldose sugar dehydrogenase to the Sup35 PrD to create three different Sup35–enzyme chimeras [77]. As in the case of the Z-domain, the fibrils were doped with the PrD alone to introduce enough space between the functional enzyme molecules to eliminate steric restrictions. The objective was to convert beechwood xylan to xylonolactone, a valuable chemical for versatile applications, in three discrete enzymatic steps. Each type of functional fibril was formed individually, and then they were mixed in different proportions to maximize the catalytic efficiency. These investigations revealed that a sequential cascade in which fibrils of xylanase A and β-xylosidase were first mixed, the resulting product of the coupled reaction removed and then incubated with fibrils of aldose sugar dehydrogenase was more productive than a mixture containing all the three kinds of fibrils together since the immobilized enzymes displayed hardly compatible stabilities. Despite its limitations, this work constitutes of nice proof of concept for prion-inspired biocatalytic cascades.

## Soft amyloid cores in PrDs and their use in nanomaterials

The absence of highly amyloidogenic sequences allows PrD to transit between soluble and aggregated states [78]. The aggregation of prions seems to depend on the establishment of a large number of weak interactions distributed along the complete PrD [79]. Accordingly, natural PrDs often consist of > 100 residues and 60 residues was traditionally considered to be the minimum length required to attain an efficient PrD self-assembly at moderate protein concentrations [46]. With this size, the PrD might constitute a significant fraction of the fusion protein, especially when it is fused to small globular domains, like the Z-domain. This results in steric restrictions in the fibrillar state since the amyloid spine may have a larger transversal dimension that the adjacent protein, a property that has been reported to reduce the diffusion of substrates and limit the functionality of the fibril[70]. Besides, large PrDs often compromise the solubility and stability of the appended globular domains in the monomeric fusions state, impacting the recombinant production of the chimeric proteins and their storage after purification.

**Figure 4.**
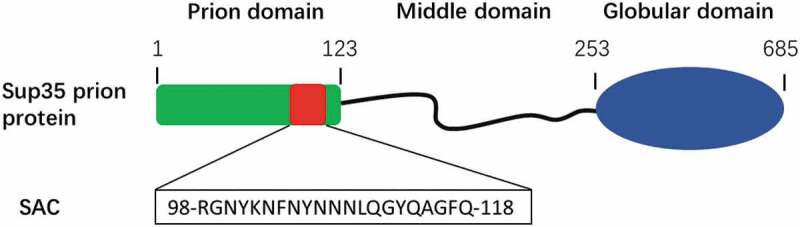
Soft amyloid core within the prion domain of Sup35p.

We have recently proposed that inner weak amyloidogenic sequence stretches within PrD might contribute to nucleate the conformational conversion of prionic proteins into a cross-β structure [80]. These soft amyloid cores (SACs) (Figure 4), were first identified in yeast prions [81], but they are also present in a significant number of human prion-like proteins [82]. SACs differ from the classical short amyloid cores of pathogenic proteins, which hold a high aggregation propensity and are typically enriched in hydrophobic residues. SACs are slightly longer and more polar, resulting in a less concentrated aggregation potential. This allows the PrD to remain soluble under most physiological conditions, but also to hold a cryptic aggregation propensity that might facilitate its efficient self-assembly in response to cellular changes [78]. The SAC from Sup35p PrD consists of 21-residues and self-assembles spontaneously into highly ordered amyloid fibrils. These fibrils can seed the amyloid formation of the complete PrD *in vitro*. Also, when the *in vitro* formed SAC fibrils are added to yeast cells, they promote the aggregation of the endogenous full-length Sup35p and the subsequent emergence of a prionic phenotype [83]. Moreover, this region is necessary for the induction, propagation, and inheritance of the prion state of Sup35p in mammalian cytosol [84]. All these data converge to suggest that this short sequence stretch might have prion-like properties, which lead us to hypothesize that SACs can substitute the role of complete PrDs and recapitulate the self-assembly properties of the larger domain in the context of modular fusion proteins.

Sup35p consists of a prion domain (green box), a charged middle domain (black line) and a C-terminal globular domain with GTP-binding activity (blue oval). The identified soft amyloid core (SAC, red box) corresponds to the indicated 21-residues long sequence.

In recent work, we genetically fused the Sup35p SAC (Sup35_SAC_, Figure 4) to three globular proteins displaying different structures, namely the all-α FF domain (FF), the all-β green fluorescent protein (GFP) and the α/β carbonic anhydrase enzyme (CA) [85]. As expected, the low aggregation propensity of the SAC allowed to express and purify the three fusions at high yields in a soluble and monomeric form, while still allowing to induce their self-assembly into amyloid fibrils under controlled conditions. As for complete PrDs, the globular proteins adjacent to the SAC retained their native structures and functions in the fibrillar state. It is worth mentioning that Sup35_SAC_-CA fibrils displayed 3-fold higher catalytic activity than that of Sup35(1-61)-CA fibrils. This increased activity likely owes to the smaller size of SAC, which would result in less steric hindrance and increased accessibility of the appended enzyme to the substrate. This study illustrated how Sup35_SAC_ could be used as a module to immobilize bioactive proteins of different sizes and structures into highly functional amyloid fibrils. The high yield at which these fusions are produced and purified and the possibility to maintain them in a soluble state until the time we want to trigger the self-assembly reaction are significant advantages relative to full length PrD-based fusions.

Importantly, not only a vast diversity of natural SACs can be identified computationally [86], but these stretches can also be artificially engineered and minimized, provided that we keep their main physicochemical traits. For instance, we have designed a family of minimalist SAC-inspired polar heptapeptides that self-assemble into amyloid fibrils. The nice thing of these nanowires is that, if they are adequately designed, they can be endorsed with intrinsic catalytic activity, without the need for an adjacent globular domain [87].

## Conclusions

In the present review, we have discussed how the genetic protein fusion strategy has resulted in the development of PrD-based functionalized nanomaterials with exceptional properties as enzyme immobilizers, biosensors, bioelectrodes or for antibody purification and antigens detection. We are convinced that the application of these unique materials in fields like nanomedicine or nanotechnology will expand significantly in the forthcoming years, especially because the use of natural and artificial SACs might allow us to potentially endorse nanofibrils with a new set of previously inaccessible functionalities.
