# Correction

**DOI:** 10.1080/19336896.2020.1804218

**Published:** 2020-08-19

**Article title**: Prion domains as a driving force for the assembly of functional nanomaterials

**Authors**: Weiqiang Wang and Salvador Ventura

**Journal**: *Cell Cycle*

**Bibliometrics**: Volume 14, Number 01, pages 170–179

**DOI**: https://doi.org/10.1080/19336896.2020.1785659

It has been noted by the authors that the reference [77] was incorrect in list. The correct reference should be ‘Schmuck B, Gudmundsson M, Härd T, Sandgren M. 2019. Coupled chemistry kinetics demonstrate the utility of functionalized Sup35 amyloid nanofibrils in biocatalytic cascades. Journal of Biological Chemistry. 2019;294(41):14966–14977’. This correction has not changed the description, interpretation, or the original conclusions of the article. The authors apologize for any inconvenience caused.

